# Degrading Ochratoxin A and Zearalenone Mycotoxins Using a Multifunctional Recombinant Enzyme

**DOI:** 10.3390/toxins11050301

**Published:** 2019-05-27

**Authors:** Md Shofiul Azam, Dianzhen Yu, Na Liu, Aibo Wu

**Affiliations:** SIBS-UGENT-SJTU Joint Laboratory of Mycotoxin Research, CAS Key Laboratory of Nutrition, Metabolism and Food Safety, Shanghai Institute of Nutrition and Health, Shanghai Institutes for Biological Sciences, University of Chinese Academy of Sciences, Chinese Academy of Sciences, Shanghai 200031, China; shofiul@sibs.ac.cn (M.S.A.); dzyu@sibs.ac.cn (D.Y.); liuna@sibs.ac.cn (N.L.)

**Keywords:** fusion enzyme, ochratoxin A, carboxypeptidase, zearalenone, ZHD101, HK293 cell, LX2 cell

## Abstract

Zearalenone (ZEA) is an estrogenic and ochratoxin A (OTA) is a hepatotoxic *Fusarium* mycotoxin commonly seen in cereals and fruits products. No previous investigation has studied on a single platform for the multi degradation mycotoxin. The current study aimed to investigate the bifunctional activity of a novel fusion recombinant. We have generated a recombinant fusion enzyme (ZHDCP) by combining two single genes named zearalenone hydrolase (ZHD) and carboxypeptidase (CP) in frame deletion by crossover polymerase chain reaction (PCR). We identified enzymatic properties and cell cytotoxicity assay of ZHDCP enzyme. Our findings have demonstrated that ZEA was completely degraded to the non-toxic product in 2 h by ZHDCP enzyme at an optimum pH of 7 and a temperature of 35 °C. For the first time, it was found out that ZEA 60% was degraded by CP degrades in 48 h. Fusion ZHDCP and CP enzyme were able to degrade 100% OTA in 30 min at pH 7 and temperature 30 °C. ZEA- and OTA-induced cell death and increased cell apoptosis rate and regulated mRNA expression of Sirt1, Bax, Bcl2, Caspase3, TNFα, and IL6 genes. Our novel findings demonstrated that the fusion enzyme ZHDCP possess bifunctional activity (degrade OTA and ZEA), and it could be used to degrade more mycotoxins.

## 1. Introduction

Mycotoxins are low subatomic weight subordinate intermediate end product of metabolism, synthesis and formed predominantly by the mycelial structure of filamentous parasites, which pose serious risks for human and animal health [1,2]. Zearalenone (ZEA) is a resorcyclic acid lactone that shows estrogenic activity in mammals generated by numerous *Fusarium* species, and it often infected cereals used for food or animal feedstuff [3]. Some investigation showed that liver and hepatic cells are the principal body parts infected by various kinds of mycotoxins. The both cell lines showed acute sensitivity: Cytoxicity and neurotoxicity induced by ZEA and OTA toxin. [4].

Ochratoxins is a subclass of mycotoxin originated from fungal genera *Penicillium* and *Aspergillus* and responsible for contaminate fruits (e.g., fruits, grains) and beverage producing farmed crops(e.g., wine, coffee) [5]. OTA is vital; a mycotoxin possesses carcinogenic, maternal pregnancy defect, immune suppressant drug, liver damage and kidney defect properties and originate through numerous fungi, including *Penicillium verrucosum, Aspergillus carbonarius*, *A. niger,* and *A. ochraceus*. These fungal species contaminate the agricultural field or storage food stuff when they obtain suitable environmental factors, like moisture and temperature [6]. Also, the liver and kidney is a major target organ for OTA; the LD_50_ range is 38–56 mg kg^−1^ for oral ingestion [7]. The total and specific cell toxicity influence of zearalenone (ZEA), aflatoxin B1 (AFB1), fumonisin B1 (FB1), and deoxynivalenol (DON) was examined over liver cells (LX2 Cells) and identified in the literature [8].

Degradation of toxins by the recombinant enzyme is an optimistic idea for the decontamination of farming products [9,10]. Food decontamination by microorganism or native enzyme is a stunning method, due to it works on targeted mycotoxin, naturefriendly, potentially efficient and generally keep good food value and without loss of nutrient and food texture [11].

Mycotoxin detoxification enzymes are naturally produced and stored intracellularly in fungi [12]. Since fungi are especially hard to lysis and extract proteins from, secreting these proteins from an *E. coli* system is especially useful in fighting mycotoxins. This recombinant bacterial secretion system will allow greater, cheaper, and faster production of these detoxification enzymes [13]. These enzymes can be applied to food with suspected mycotoxin existence, or food that has already been affected, to eliminate the toxic threat. The purified fusion recombinant enzyme can also be applied directly to crops or food products [14]. Thus, this bifunctional enzyme plays an indirect yet key role in the regulation of mycotoxin control, especially for cereals and agricultural grain products. We will mainly focus this study on recombinant enzymes for degrading *Aspergillus* and *Fusarium* mycotoxins in cereal derived foods and feeds.

Literature from previous studies suggests that some natural enzyme degrades only single mycotoxin contaminate cereals and fruits products [15]. However, no one report about bifunctional or multifunctional recombinant enzyme activity for degrades different enzyme. We hypothesize that fusion and recombinant genetic engineering of two single enzymes can be more advanced and superior than single and have the ability deteriorates several mycotoxins, that can minimize the mycotoxin load and produce enzymatic process some final nontoxic products. It is anticipated that we will use some bacterial cloning system to established detoxification system and validation of the existing system. As a change of the mostly used fusion gene development technique, this technique may additionally find many applications in bioscience, bioengineering, and molecular biotechnology.

ZHD101 is a single recombinant enzyme that degrades ZEA [16]. OTA degradation by carboxypeptidase from *B. amyloliquefaciens* ASAG [17]. Fusion proteins are appointed in biochemical and biophysical practice, for example enzyme purification, protein crystallization, drug discovery, trigger of prodrug, apply in antibody preparation and immune physiological drug delivery solution [18,19]. The bifunctional enzyme defined as an enzyme is an enzyme carry two unique catalytic abilities in the similar polypeptide chain [20,21]. We fused ZHD101 and carboxypeptidase enzyme to fusion enzyme ZHDCP and expressed using mobile as a single protein. Fusion protein is a output of a recombinant DNA technology, its defined as a novel class of biocompound with numerous action properties. Combining two or more protein subjects together by genetic engineering technology, it was found that the fusion protein characterized several functional properties gathered from each parent protein subject [22].

Furthermore, the study aimed to discover the mechanism and biochemical characterization of recombinant enzyme detoxification activity. 

## 2. Results

### 2.1. Expression and Purification of ZHDCP, ZHD101 and Carboxypeptidase Enzyme

**Figure 1:**
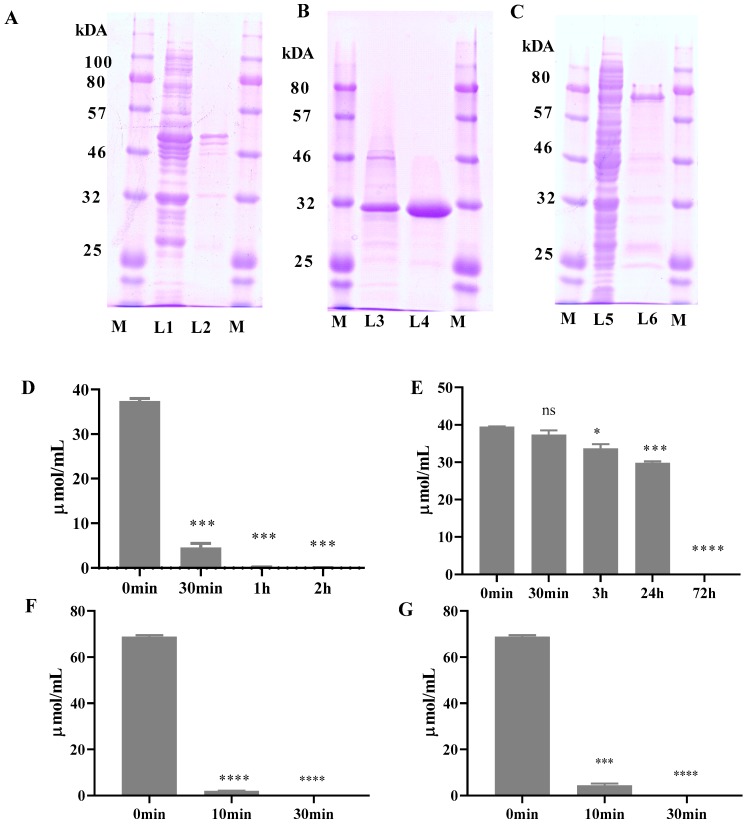
Enzyme expression, purification and degradation efficiency. (**A**) SDS-PAGE gel staining of fusion enzyme. Where, M: protein marker, L1: Carboxypeptidase crude protein, L2: Carboxypeptidase Ni-NTA purified protein (48.66 kDa), (**B**) L3: zearalenone hydrolase 101 (ZHD101) crude protein, L4: ZHD101 Ni-NTA purified protein (28.7 kDa), (**C**) L5: Fusion enzyme (ZHDCP) crude protein, L6: Fusion enzyme Ni-NTA purified protein (77.36 kDa). (**D**) ZEA (50 µM) degradation by ZHDCP enzyme, (**E**) ZEA (50 µM) degradation by carboxypeptidase enzyme, (**F**) OTA (50 µM) degradation by ZHDCP enzyme, (**G**) OTA (50 µM) degradation by carboxypeptidase enzyme. The error bars represent the standard deviation. *, ***, **** indicates a significant difference between control (0 min) and ZEA/OTA degradation by ZHDCP or Carboxypeptidase enzyme in different time at *p* < 0.05, *p* < 0.001, and *p* < 0.0001. ns: not significant.

Results presented in Figure 1A showed that carboxypeptidase and ZHD101 (Figure 1B) sequences were fused in pET28a vector map by a double polymerase chain reaction. The pET28a vector has a T7 promoter region that facilitated Bl21 (DE3) expression. The recombinant induced protein migrated with an apparent molecular weight, on sodium dodecyl sulphate-polyacrylamide gel electrophoresis(SDS-PAGE) gel staining carboxypeptidase was showed 48.66 kDa, ZHD101 (28.7 kDa) and fusion ZHDCP enzyme (77.36 kDa) (Figure 1C) almost their estimated protein size. For purification of fusion enzyme zearalenone hydrolase-carboxypeptidase (ZHDCP) and single recombinant enzyme, lysis protein was bound with Ni-NTA resin. In the crude extract of *E. coli* cells, we were able to distinguish very weak ZEA and OTA degradation activity using the above experimental environment. The elution protein then underwent dialysis by low NaCl and without no imidazole to make protein stable condition and pH of the protein, which was 7.5. Cloning of fusion ZHDCP enzyme sequences, ZHD101, and carboxypeptidase on pET28a vector in Bl21 (DE3). This finding was supported by restriction digestion with (SacI, HindIII) and DNA sequences, which verified the identity of the construct.

### 2.2. ZEA Degradation by ZHD-CP Enzyme

**Figure 2:**
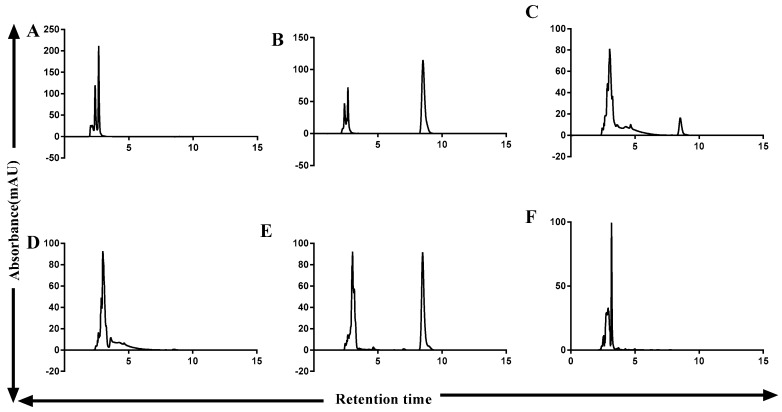
Detection of ZEA degrading efficiency of ZHD-CP & carboxypeptidase enzyme by Ultraviolet-High performance liquid chromatography (UV-HPLC). (**A**) Blank(Buffer), (**B**) Standard ZEA 50 µM, (**C**) ZEA 50 µM with ZHDCP in 30 min, (**D**) ZEA 50 µM with ZHDCP in 2 h, (**E**) ZEA 50 µM with carboxypeptidase in 30 min, (**F**) ZEA 50 µM with carboxypeptidase in 72 h.

Purified fusion ZHDCP enzyme containing 100 µg of enzyme can degrade 50 µM ZEA within 2 h and its conversion efficiency is 100% (Figure 1D,E). We found that Ultraviolet-High performance liquid chromatography (UV-HPLC) detection of ZEA retention time is 8.15 min (Figure 2) and a sharp peak of ZEA by UV-HPLC at 274 nm wavelength. For ZEA degradation fusion ZHDCP, carboxypeptidase and ZHD101 enzymes optimum temperature are 35 °C, 35 °C, 40 °C and optimum pH 7.0, 7.0, 8.0, respectively, presented in Table 1 and Table 2.

### 2.3. OTA Degradation by Fusion (ZHDCP) Enzyme

**Figure 3:**
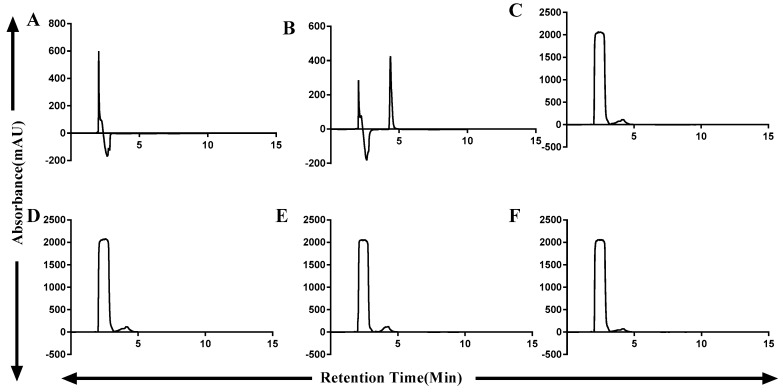
Detection of OTA degrading efficiency of ZHDCP and Carboxypeptidase enzyme by UV-HPLC. (**A**) Blank (Buffer), (**B**) Standard 50-µM OTA, (**C**) 50-µM OTA treated with ZHDCP enzyme in 30 min, (**D**) 50-µM OTA treated with ZHDCP enzyme in 24 h, (**E**) 50-µM OTA treated with carboxypeptidase enzyme in 30 min, (**F**) 50-µM OTA treated with carboxypeptidase enzyme in 24 h.

50 µM OTA degradation by 100 µg of the purified enzyme showed 100% OTA degradation in 30 min (Figure 1F,G). We found that UV-HPLC detection of OTA retention time was 4.13 min in Figure 3. We also detected OTA detection by UV 214-nm wavelength, where most of the previous studies used HPLC-FLD system. ZHDCP enzyme was optimum at the temperature of 25 °C and pH 7. OTA degradation by carboxypeptidase was at a temperature of 35 °C and pH 7 (Table 1 and Table 2).

### 2.4. ZEA Degradation Products by Fusion Enzyme

**Figure 4:**
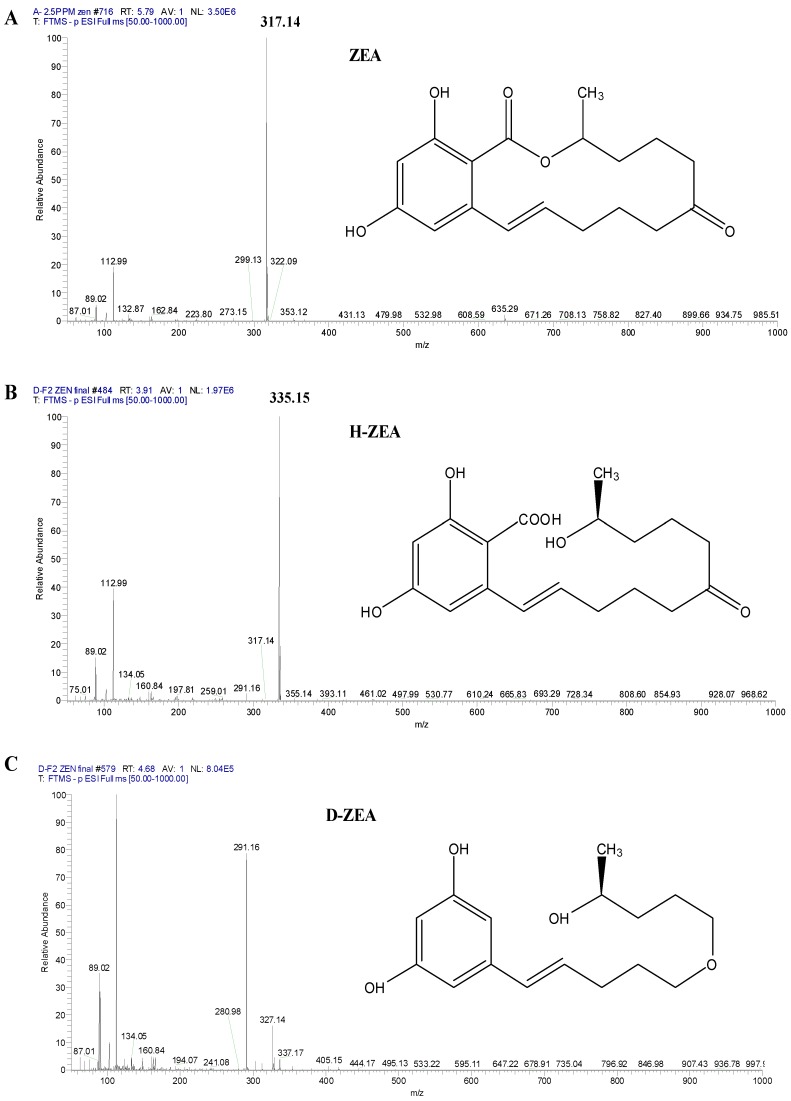
ZEA and ZEA degradation final products by ZHDCP and carboxypeptidase enzyme. (**A**) Electrospray ionization (ESI)(+)-MS spectra of ZEA: *m*/*z* 317.14. (**B**) ESI(+)-MS spectra of ZEA degradation product H-ZEA: *m*/*z* 335.15. By ZHDCP degradation, (**C**) ESI(+)-MS spectra of ZEA degradation product D-ZEA: *m*/*z* 291.16.

It can be seen from Figure 1D, showed that fusion ZHDCP could degrade completely in 2 h to the non-harmful product. From Figure 2, it was observed that carboxypeptidase could degrade ZEA in 8 h to complete 100% conversion to nontoxic product. Still, there was a need to check in high resonance spectroscopy to confirm the presence of non-harmful ZEA degrade product. In addition, we needed to do the cell apoptosis; cytotoxicity and cell viability test to the final product to ensure no harmful effect on the human body. We used 50 μg of the purified and concentrated enzyme for incubation with the toxin. We also used the non-purified enzyme for ZEA degradation. Then, it was observed that only a purified enzyme has a mycotoxin (ZEA) detoxification ability. Nevertheless, the non-purified enzyme has less degradation activity. We also used an empty vector and buffered with the toxin. However, we observed that the empty vector and reaction buffer had no degradation activity. We identified a less harmful reproductive metabolite (1-(3,5-dihydroxy-phenyl)-10′-hydroxy-1′-undecen-6′-one) than ZEA [23]. Thereafter, we kept homogenization by mixing with the ultrasonification of the purified enzyme with ZEA, and identified the earlier elusive principal reaction metabolite hydrolyzed ZEA (H-ZEA) by liquid chromatography-tandem mass spectrometry, purified it by preparative high-performance liquid chromatography, and confirmed its proposed structure ((E)-2,4-dihydroxy-6-(10-hydroxy-6-oxo-1-undecen-1-yl)-benzoic acid) by nuclear magnetic resonance systems. In the extemporaneous decarboxylation to D-ZEA ((E)-1-(3, 5-dihydroxy-phenyl)-10-hydroxy-1-undecen-6-one), we noticed an earlier suggested isomer in Figure 4 [15]. α-zearalenol, β-zearalanol, β-zearalanol was not identified, but detected by mass spectrometry in this investigation as shown in Table 3 [24].

### 2.5. OTA Degradation Products Convert by Fusion Enzyme and Carboxypeptidase Enzyme

**Figure 5:**
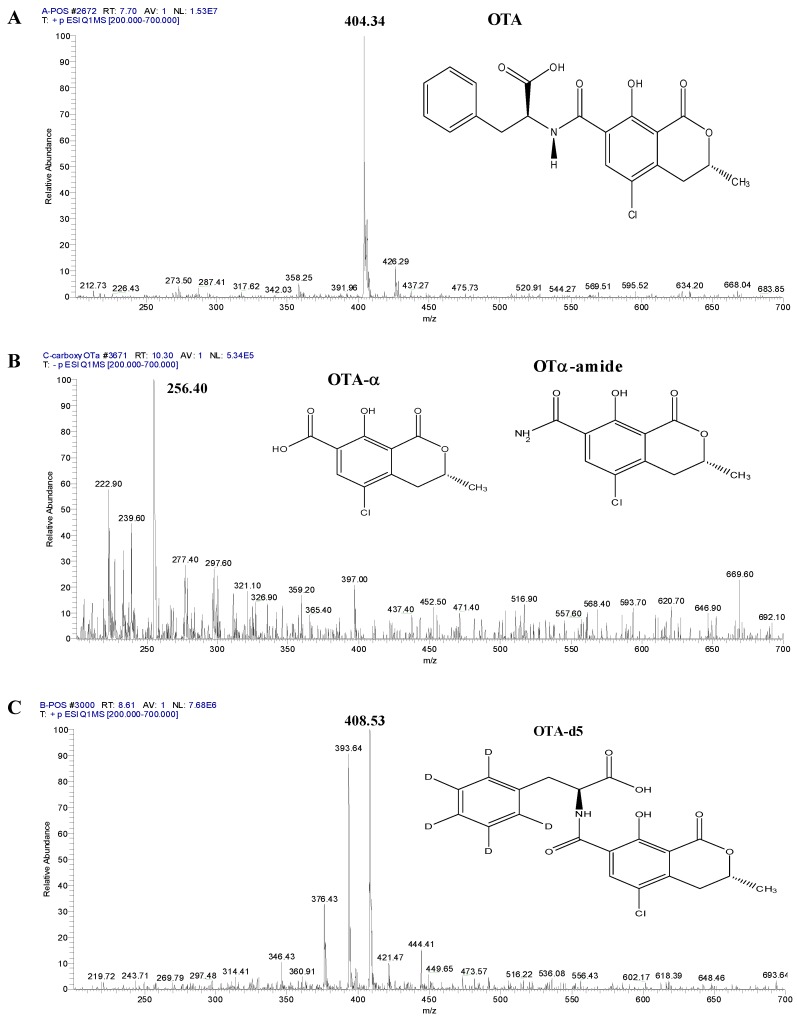
OTA and OTA degradation final products by ZHDCP and Carboxy enzyme. (**A**) Electrospray ionization (+)-MS spectra of OTA: *m*/*z* 404.34, (**B**) Electrospray ionization (+)-MS spectra of ochratoxin α amide: *m*/*z* 256.40. OTA degradation by ZHDCP enzyme, and electrospray ionization (+)-MS spectra of ochratoxin α: *m*/*z* 239.60 by Carboxy enzyme degradation, (**C**) Electrospray ionization (+)-MS spectra of ochratoxin A-d5: *m*/*z* 408.53.

Ochratoxin-α (OTα) is a vital degradation final product of OTA and has been recognized to take place in cultures of OTA generations species. Mammals are infected by OTA from small doses of poisonous toxin from a different variety of foods [25]. OTA degradation by both ZHDCP and carboxypeptidase enzymes have almost similar degradation products. The main degradation products found in this study were OTAα (239.60 MW), ochratoxin α amide (256.40 MW) and ochratoxin A-d5 (408.53 MW) as shown in Figure 5. Studies from other researchers showed OTα and OTα-amide degradation from OTA [20]. However, the breakdown of OTA to the nontoxic OTα, hydrolyzed by a carboxypeptidase, was identified, and the kinetic report on OTA were clarified [26]. It can be inferred that the derived metabolites of OTA were hydrolyzed into OTα by carboxypeptidase produced by ASAG1. Microbes and natural biotransformation enzyme degradation methodologies were required to derive sustainable and alternative biocontrol. In the future, more experimental study is needed to determine if more dominant substances should be used in a decontamination process in order to identify whether any other active substances play different roles in the detoxification process [27].

### 2.6. ZEA and OTA Trigger of Cell Viability Inhibition

**Figure 6:**
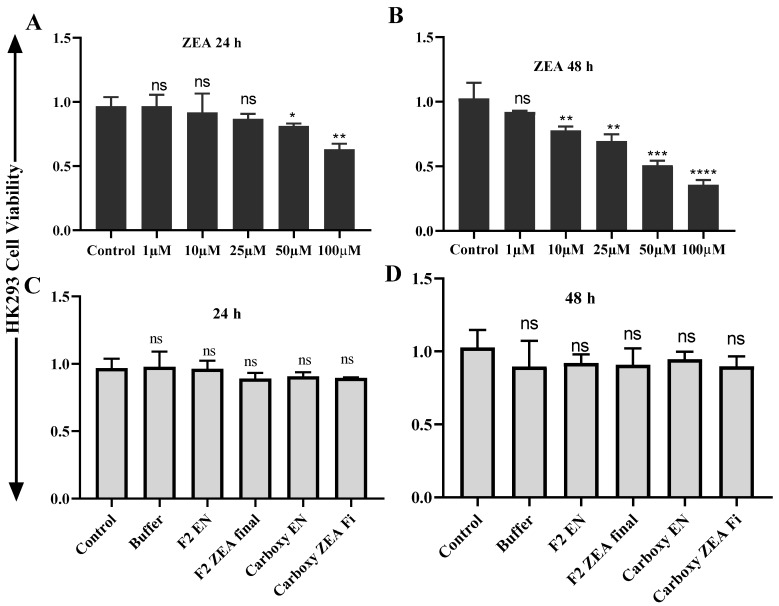
ZEA cell cytotoxicity test on HK293 and its final products. (**A**) Effect of ZEA on HK293 cell at 24 h, (**B**) Effect of ZEA on HK293 cells on 48 h, (**C**) Effect of ZEA final products on HK293 cell in 24 h, (**D**) Effect of ZEA final products on HK293 cells in 48 h. The values are mean ± SD of three independent experiments.*, **, ***, **** indicates a significant difference between ZEA and control at *p* < 0.05, *p* < 0.01, *p* < 0.001, and *p* < 0.0001. ns: not significant.

**Figure 7:**
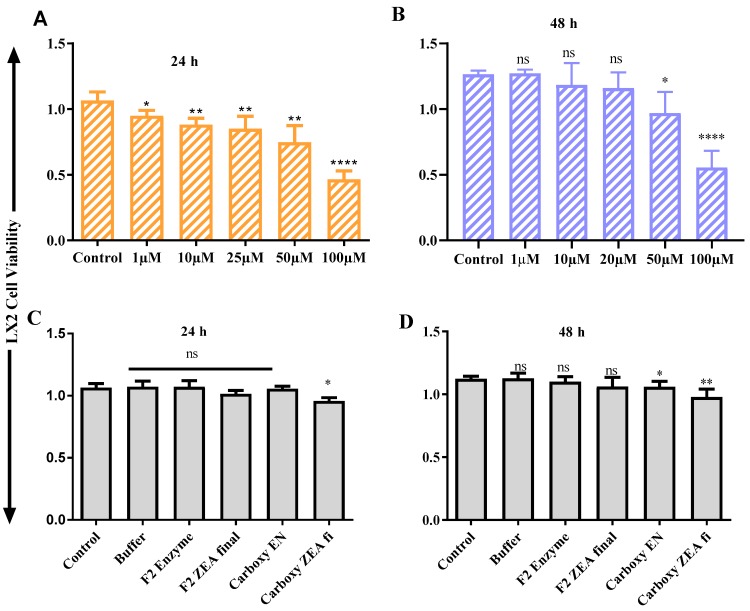
In vitro cell toxicity test ZEA on liver cells (LX2). (**A**) ZEA effects on LX2 cells in 24 h, (**B**) ZEA vs. LX2 cells in 48 h, (**C**) LX2 vs. ZEA final products in 24 h, (**D**) LX2 vs. ZEA final product in 48 h. Each set of data shows the mean ± SD of the three independent experiments. *, **, ***, **** indicates a significant difference between ZEA and control at *p* < 0.05, *p* < 0.01, *p* < 0.001, and *p* < 0.0001. ns: not significant.

**Figure 8:**
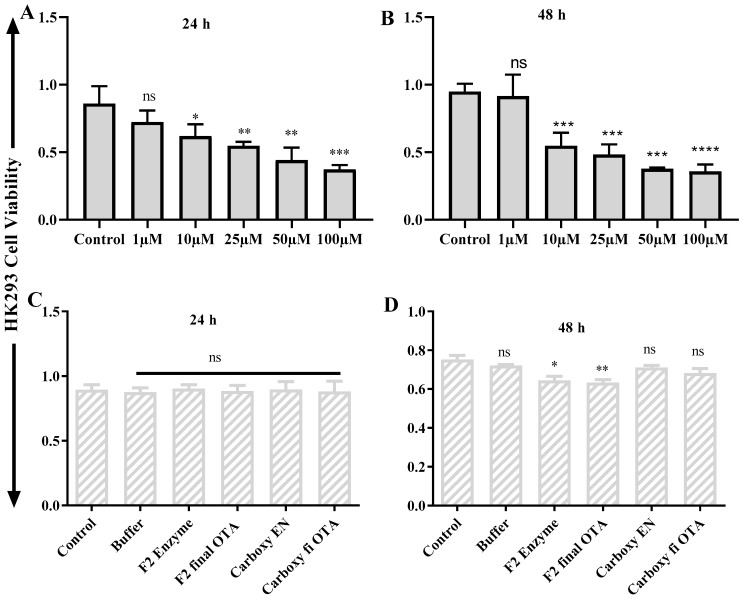
OTA cell cytotoxicity test on HK293 and its final products. (**A**) Effect of OTA on HK293 cells at 24 h, (**B**) Effect of OTA on HK293 cells on 48 h, (**C**) Effect of OTA final product on HK293 cells in 24 h, (**D**) Effect of OTA final product on HK293 cells in 48 h. Each set of data shows the mean ± SD of the three independent experiments. *, **, ***, **** indicates a significant difference between OTA and control at *p* < 0.05, *p* < 0.01, *p* < 0.001, and *p* < 0.0001. ns: not significant.

**Figure 9:**
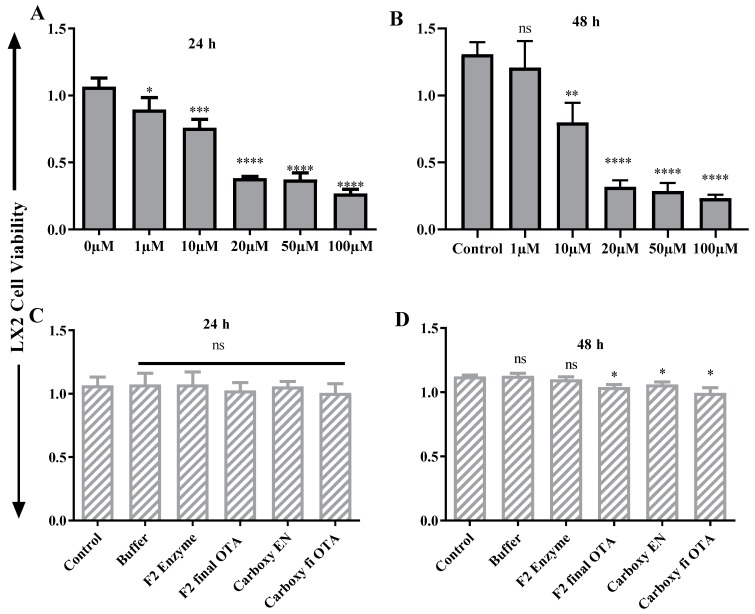
In vitro cell toxicity test OTA on liver cells (LX2). (**A**) OTA effects on LX2 cells at 24 h, (**B**) OTA vs. LX2 cells at 48 h, (**C**) LX2 vs. OTA final product in 24 h, (**D**) LX2 vs. OTA in 48 h. Each set of data shows the mean ± SD of the three independent experiments. *, **, ***, **** indicates a significant difference between OTA and control at *p* < 0.05, *p* < 0.01, *p* < 0.001, and *p* < 0.0001. ns: not significant.

The cell toxicity consequence of ZEA and OTA on hepatic (HEK293)/ liver (LX2) cells succeeding 24 h/48 h incubation carried out by the Cell Counting Kit-8 (CCK-8) test are presented in Figure 6, Figure 7, Figure 8 and Figure 9. After 24 h/48 h of incubation, ZEA/OTA treatment with experimental dose varying from 1–100 μM created a remarkable decline of cell viability in a dose-dependent mode, and the IC_50_ values for ZEA were 50 μM. Regarding the impact of ZHDCP treatment on cell viability, a high concentration (>10 μM) reduced the cell viability while no destructive outcome was observed under a concentration of 10 μM. For the 50 μM ZEA treatment with Hk293 cell lines for 24 h, we observed a significant (*p* < 0.01) inhibition. For the 48 h, when treated with the HK293 cell, the ZEA 10 μM dose shows significant cell inhiation and cell death due to acute toxicity in the culture media. For the LX2 cell lines, when treated with ZEA, from 1 μM to 100 μM, all doses shows a significant amount of cell death. A smaller concentration of ZHDCP initiated the cell proliferation rate, making clear its favorable consequences on cell growth and its potential to antagonize the effect caused by mycotoxin ZEA. However, co-treatment by both ZHDCP and ZEA did not show any beneficial effect on cell growth, but pretreatment of cells by ZHDCP showed its protective effect on cell proliferation inhibition caused by ZEA. Thus, a method of ZHDCP pretreatment was used in the following experiments to evaluate the protective effect of ZHDCP on ZEA-induced cytotoxicity. The chemical structure and synthesis of ZEA are similar to natural estrogen [28]. ZEA can competitively bind to the estrogen receptor, causing external and internal genitals with alterations and reproduction disorders. Therefore, humans and animals are easily affected by the ZEA, in which ZEA usually enters the food chain through the toxicated cereals and stored in human and animal bodies. Findings from previous research work have determined that ZEA proceeds to hyperestrogenism symptoms and in excess, affects human reproduction [3].

### 2.7. Protective Effect of Fusion ZHDCP and CP Enzyme Final Products on the Cell Life and Surviving

As interpreted and elucidated in Figure 6C,D, Figure 7C,D, Figure 8C,D and Figure 9C,D, ZHDCP and CP final products significantly reduced the ZEA/OTA exposure, and it has shown that those final products have no significant cell death effect in the in vitro cell cytotoxic study, whereas the ZEA/OTA dose-dependent manner shows significant cell death effect. From this investigation, it also showed that individual single enzymes and degradation reaction buffer has no significant effect on cell viability. Therefore, we conclude from this experiment that these two enzymes and the final enzymatic product have a less cytotoxic effect. Also, ZHDCP and a 100-μg CP concentration to degrade 50 μM ZEA/OTA significantly promoted or recovered the cell viability. The extra ZHDCP and CP enzyme could be retained on the cell walls and after that, its absorption in the subsequent cell culture medium containing ZEA improved the amount of cellular ZEA ingestion. For that reason, the ZEA/OTA experimental dose was select to be 50 μM in the subsequent test, as ZEA and OTA at this amount created a prominent decline of cell viability and the cell injury properties would be diminished by the ZHPCP enzyme.

### 2.8. Effect of Fusion (ZHDCP) Enzyme Final Products on ZEA/OTA Induced Cell Apoptosis

**Figure 10:**
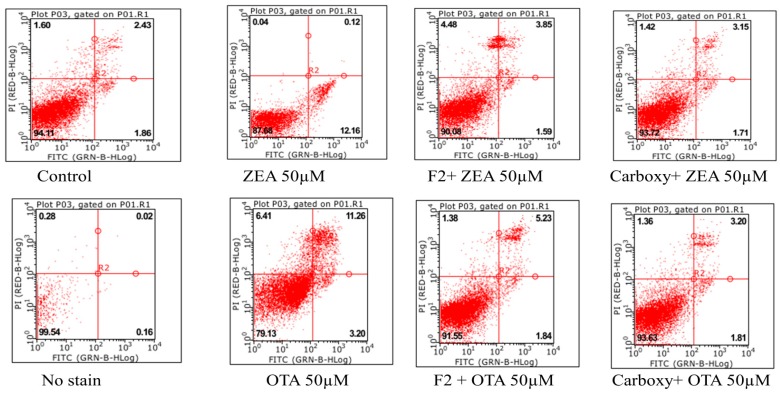
Effect of fusion of the ZHDCP and CP enzyme final products from ZEA- and OTA-reduced apoptosis in LX2 cells. In the figure, cell apoptosis rate is shown in terms of %.

**Figure 11:**
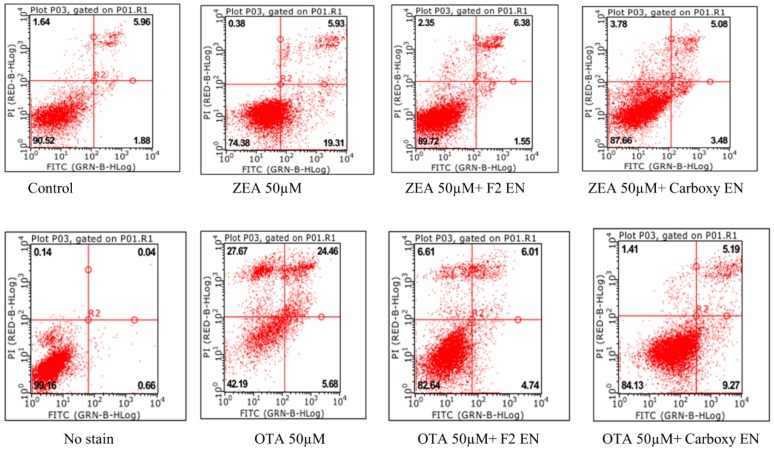
Effect of fusion ZHDCP and CP enzyme final products from ZEA- and OTA-reduce apoptosis in HK293 cells. Annexin V-FITC/PI flow cytometry was used to detect HK293 cells treated with ZEA (50 µM) and OTA (50 µM). The Q1, Q2, Q3, and Q4 gates, respectively, symbolized dead cells, the late stage of cell apoptosis, normal cells, and the early stage of cell apoptosis (A, B, C, D, and E are control, ZEA 50 µM, ZEA 50 µM + ZHDCP 100 µg, ZEA 50 µM + Carboxy 100 µg, 50-µM OTA, 50-µM OTA + 100 µg ZHDCP enzyme, 50-µM OTA + 100 µg CP enzyme, respectively).

The finding from Figure 10 (LX2) and Figure 11 (HK293) explain cell mitochondrial apoptosis interaction with ZEA 50 μM, OTA 50 μM, and fusion ZHDCP and carboxypeptidase enzymatic degradation final products. It’s meaningful that flow cytometry depended on the double-staining reagents of PI and Annexin V-FITC intact cells (FITC−/PI−) as well as early apoptosis (FITC+/PI−). Late apoptosis/necrotic cells (FITC+/PI+) elucidated that ZEA significantly increase the apoptosis rate; the ZEA 50-μM dose induced apoptosis 8.39% and 17.41% for LX2 and HK293 cells, respectively.

However, 50 μM of OTA induced 10.17% (LX2) and 22.3% (HK293) apoptosis level, where the control group apoptosis level was 4.29%. It has been noticed that the final products reduce significantly at the treated cell-line apoptosis level. For ZEA final products produced by ZHDCP and CP, the enzyme apoptosis level reduced by 7.24% and 7.82%, respectively; for the LX2 cell and for the HK293 cell, 17.31% and 16.68% respectively, which is statistically significant. When OTA degradation products produced by ZHDCP and CP for LX2 cell (7.93%, 9.39%) and HK293 cell (19.39%, 15.68%), the apoptosis level reduces, meaning these final products have no harmful health effects. For the reason consistence susceptible of cells to ZEA/OTA, the fragmented redox balance and reduce of mitochondrial membrane potential would eventually lead to the appearance of apoptosis. was Also, there is evidence in the study that ZHDCP released the ZEA-induced cell cycle effect; we hypothesized that ZHDCP could also inhibit the apoptosis caused by the OTA. After pretreatment by ZHDCP, the apoptosis rate under 20-μM ZEA treatment was decreased significantly to the control level. In addition, cell apoptosis initiated by 50-μM ZEA was only partly recuperated by pretreatment of ZHDCP enzyme. Precisely, apoptotic cells in both early (4.25% and 6.18%) and late (4.70% and 8.10%) stages were increased after ZEA treatment, and ZHDCP pretreatment mainly reduced the apoptotic cells in their early stage. Similarly, there were less luminously stained and splitted the shape of the cells in ZHDCP pretreatment groups, which means less apoptosis compared to the ZEA/OTA treatment groups.

### 2.9. The Mycotoxin Effect on mRNA Gene Expression Induced by Necrobiosis

**Figure 12:**
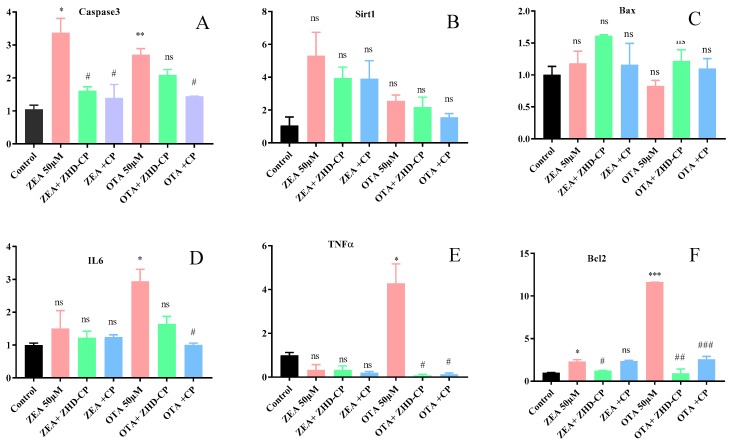
The mRNA expression level of apoptosis gene by ZEA (50 µM), OTA (50 µM) and their enzymatic degradation final products. (**A**) Caspase3, (**B**) Sirt1, (**C**) Bax, (**D**) IL6, (**E**) TNFα, (**F**) Bcl2 mRNA expression level in the Lx2 cells. Cells were exposed to ZEA/OTA and enzymatic degradation derivatives for 24 h. The results are expressed relative to the expression of β-Actin; each set of data shows the mean ± SD of the three independent experiments. *, **, *** indicates a significant difference between ZEA, OTA and control at *p* < 0.05, *p* < 0.01, and *p* < 0.001. ns: not significant. #, ##, ###, indicates a significance difference between ZEA/OTA compare with their enzymatic final products at *p* < 0.05, *p* < 0.01, and *p* < 0.001.

ZHDCP showed a mycotoxin biotransformation property and it’s also visible that ZHDCP could initiate SIRT1, via a direct or indirect pathway in both in vitro and in vivo studies. SIRT1 regulates a variety of processes that could alter cell response to toxicant-induced cytotoxicity, including the detoxification of reactive oxygen species (ROS) by the upregulation of MnSOD, DNA repair activation (cyclin D, GADD45, p27/Kip1) and resistance to apoptosis. From the outcomes, it has been understood that ZHDCP attenuated ZEA/OTA-induced oxidative damage and apoptosis. In order to further determine the protective mechanism of ZHDCP on cells, expression of SIRT1 and apoptosis-related genes of Bax and Bcl2 were analyzed using quantitative real-time PCR after treatment by ZEA 50 μM/OTA 50 μM in the absence and the presence of ZHDCP/CP final products’ pretreatment. Bax and Bcl2 are apoptosis cofactor in which Bcl2 suppresses apoptosis by controlling the mitochondrial membrane permeability and preventing the release of cytochrome-C from mitochondria while Bax antagonizes Bcl2 by translocation to mitochondrial membrane proceedings to cytochrome-C discharge and apoptosis. As illustrated in Figure 12, ZEA (1.44 ± 0.15) itself initiated prominent expression of Bax while there is no significant difference in the expression level of SIRT1 and Bcl2 between the ZEA and control groups (∼1). From the finding of the mRNA expression study, we found that when 50-μM ZEA induced the following genes to fold: Caspase3 (2.32 ± 0.31↑), Sirt1 (4.25 ± 0.91↑), Bax (0.18 ± 0.06↑), IL6 (0.51 ± 0.1↑), TNFα (0.67 ± 0.12↓), Bcl2 (1.32 ± 0.20↑). However, the 50-μM OTA treatment in the LX2 cell, the following gene folds changed in comparison to the control group: Caspase3 (1.65 ± 0.06↑), Sirt1 (1.49 ± 0.0.15↑), Bax (0.17 ± 0.05↓), IL6 (1.95 ± 0.3↑), TNFα (3.29 ± 0.75↑), Bcl2 (10.62 ± 0.01↑). Whereas, ZHDCP individual created a high expression of SIRT1 (6.79 ± 0.54) and Bcl2 (1.55 ± 0.21) and low expression of Bax. In the ZHDCP+ZEA group, the expression of Bax was inverted to the control level, but the expression of SIRT1 (3.28 ± 1.58) and Bcl2 (1.51 ± 0.18) was still higher than the control. 

Furthermore, the mRNA level of Bcl2 in the ZEA treated group was found to be high, even though it is not statistically significant than the control group, which might be as the result of stress resistance in cells. The ratio of Bax to Bcl2 was also significantly reduced from ZEA (∼1.23) to the ZHDCP+ZEA group (∼0.75), indicating an inhibition of the mitochondria-mediated apoptosis. A vital notice was that ZEA treatment significantly declined the ZHDCP pretreatment and induced a high expression of SIRT1 from 6.79 ± 0.54 to 3.28±1.58, which also showed the cytotoxicity of ZEA that can deleterious consequences of the gene expression. Findings showed that mRNA level of Bax, Caspase3 increases, but, SIRT1, TNFα, IL6, and Bcl2 decreases by ZEA/OTA treatment.

## 3. Discussion

We defined and characterized a novel fusion enzyme named ZHDCP and its total design and protein expression in a lab scale in our current investigation. It has been observed that the fusion enzyme ZHDCP and the mono enzyme carboxypeptidase—in addition to removing ZEA/OTA in the reaction buffer—can also degrade ZEA in the infected crops. Further work is essential and need to be done to know the proper method and the ZEA/OTA control measures of agricultural cash crops.

It is very important to protect from OTA generation by controlling fungal breeding and outbreak. In addition, these control procedures are hard to establish with the ultimate result of OTA in the final farming products. Preventive strategies are frequently applied to removal, due to limitations and to avoid the poisonous effect of OTA. Biological strategies were approved internationally instead of chemical and physical control measures. Belsare et al. have discussed the fusion protein generation process [29]. However, examples of practical purposes are limited. A significant ratio of the global plant-based meals is toxicated with fungal mycotoxins, and more effective and safer strategies for detoxification and remediation are required. Enzymes could be a realistic path to eradicate the intensity and to keep away the poisonous consequences by way of bioremediation [30,31].

OTA biodegradation by carboxypeptidase was inhibited by several chelating substances for example 1,10-phenanthroline and ethylenediaminetetraacetic acid, concluded that carboxypeptidase is a metalloprotease, uniform identity with carboxypeptidase A, which was produced by *P. rhodozyma* [32]. The purified fusion engineered recombinant enzyme was mixed and store with ZEA or OTA toxin, the amount of ZEA or OTA was significantly and quickly degraded compared to the previous report. OTA is a colorless crystal compound of empirical formula C20H18O6NCl, and its molecular weight of 403.822 kDa [33]. The maximum allowable limit of OTA occurrence is 20 ppb and its safe limit is recognized by the national and international criteria for apples and grapes [34]. The complete biotransformation of ZEA has not been investigated, however the biological degradation of ZEA to zearalenols has been studied, which is recognized as more estrogenic than the mother metabolite [35]. ZEA degradation by an enzyme activity mechanism needs to be elucidated, as it is a significant issue. The ZHD101 hydrolase enzyme origin from *Clonostachy rosea* is accountable for the ZEA detoxification [16] and could be discovered as an effective control method for the security of cereals from ZEA toxification. Biotransformation of ZEA does not determine complete detoxification of ZEA, due it may produce the more estrogenic metabolites: α-zearalenol, α-zearalanol, β-zearalanol [24]. It is important to note that ZEA toxification by ZHDCP and carboxypetidase enzyme produce H-ZEA and D-ZEA, which were recognized as less estrogenic derivatives. 

This study demonstrated that ZEA and OTA toxin-induced cell death in human Hk293 & LX2 cell proceeds primarily via apoptosis. ZEA obstructs amino acid and DNA production and starts lipid peroxidation and programmed cell death [36]. Cell apoptosis defined as a branch cell toxicity sciences, where cell death is preplanned and organized, and is a vital system in cell development, cell multiplication. It is also self-regulating and it is remarked by conformation modification, for example, the breakage of DNA structure in eukaryotes and the development of caspase-mediated cell death substances [37,38]. From cell cytotoxicity assay, OTA showed more toxicity than ZEA. Additionally, ZHDCP fusion enzyme treated mycotoxin final products were found to reduce cell death and inhibited the apoptosis induced by ZEA and OTA. Cell apoptosis occurred due to contact with mycotoxins, mitochondrial-facilitated apoptosis is the fundamental pathway, which is initiated programmed cell degeneration by reactive oxygen species and facilitated by mycotoxins [39]. This study assists scientists to explore new biological markers for ZEA and OTA breakdown and offers additional perceptions to the molecular and cellular mechanisms of ZEA and OTA. 

## 4. Conclusions

Considering the serious problems, such as diseases resulting from *Fusarium* and *Aspergillus*, which frequently occur in cereals, wheat, fruits and other agricultural products worldwide, ZEA and OTA infection of crops is an important issue to be considered. In this study, we have cloned, expressed and purified a novel fusion enzyme liable for biotransformation of ZEA and OTA in food and feed products. From the data provided by the literature and current research showed that OTA and ZEA are severely poisonous, but their presumptive degradation metabolite, OTA (OTα, OT-d5, and OTα-amide) and ZEA (HZEA, DZEA) are reported as not toxic. An in vitro model (HK293 & LX2 cells) study showed that the fusion enzyme’s final products have less cytotoxicity and reduce mitochondrial apoptosis induced by OTA and ZEA. From the current research, it can be summarized that the mono and fusion recombinant enzymes of ZHDCP are a possibility for use in the field and and industrial applications of a bio-transformation strategy and innovative tool. This bifunctional purified enzyme is more efficient and fast compared to the strain that degrades mycotoxin. Further investigations are required to unveil the fusion enzyme multifunctional activity test on another mycotoxin (AFB1, DON, FUM, etc.). This fusion system can later be applied directly in the field (fruits and cereals products).

## 5. Materials and Methods

### 5.1. Chemicals

Restriction enzymes, T4 DNA ligase, DL5000 DNA markers, and DNA loading buffer were from Takara, plasmid extraction kits from Tiangen Biotech Co. Ltd. (Beijing, China), and gel extraction kits were bought from Axygen Biosciences (Union City, CA, USA. Protein markers from Cell Signaling Technology (Danvers, MA, USA), Trans 2xnEasy Taq PCR Super Mix, 4S Green Plus Nucleic acid stain, protein loading buffer from Yeasen Biotechnology Co. Ltd. (Shanghai, China), SDS gel stain wash buffer from Beyotime Biotechnology Co. Ltd. (Shanghai, China), R250 from Ourchem, Sinopharm Chemical Reagent Co. Ltd. (shanghai, China), Ni-NTA from TransGen Biotech Co. Ltd., (Shanghai, China). pET28a from Novagen (Foster City, CA, USA), PMD18T, Takara Co. Ltd. (Dalian, China). 

### 5.2. Standard

Ochratoxin A standard was purchased from Romer Lab (Getzersdorf, Austria). Zearalenone was bought from Sigma (Shanghai, China) and made 1 mg/mL stock solution in methanol solvent in this current research investigation. Then this stock solution was then diluted with HPLC grade methanol to achieve the suitable work solutions. Before HPLC analysis of the sample and standard, OTA and ZEA solutions were preserved and refrigerated in darkness at −20 °C. Acetonitrile, methanol, water, ethyl acetate (everything were HPLC purity) and acetic acid were purchased from ANPEL laboratory Technologies, Shanghai, China. 

### 5.3. Construction of Fusion Gene (ZHD101+ Carboxypeptidase) and Expression in E. coli

The single gene zearalenone hydrolase from *Clonostachys rosea* strain IFO7063 (Accession No: AB076037) [16] and carboxypeptidase gene from *Bacillus amyloliquefaciens* strain ASAG1 (Accession number: KP161493) [40] sequence are synthesis and cloned into pUC57-Amp vector by Genewiz Company, Beijing, China. The expression vector for the fusion enzyme (ZHDCP) was constructed through PCR using four synthetic primers (Table A1). First primer (ZHD-F, ZHD-R) and (Carboxy-F, Carboxy-R) and corresponding template (50–100 ng) are amplified through PCR. Then two PCR products were purified, mixed and subjected to PCR using the primers ZHD-F and Carboxy-R, and the amplified (ZHDCP) fusion gene was cloned into a pET28a vector. The plasmid was purified from a positive clone for transformed into *E. coli* BL21 (DE3) as a His_6_ fusion protein.

### 5.4. Protein Expression and Purification of ZHD101, ZHDCP, and Carboxypeptidase Enzyme in Heterologous Hosts

Luria Bertani (LB broth) (Tryptone—10 g, Yeast extract—5 g, NaCl-10 g, Milli-Q water—1 L) was autoclaved at 121 °C for 20 min. Dilute a 50-mL overnight culture 100× into pre-warmed media (9 mL to 900 mL). Grow at 37 °C for 4~6 h till OD_600_ reaches 1~(0.6), induced with 0.5 mM IPTG and shake at 16 °C for overnight (~16 h). The expression cells were collected and disrupted by sonication (Ultrasonic Homogenizer JY92-iiDN, Ningbo Scientz Biotechnology Co. Ltd., Ningbo, China). Lysis buffer contained 10 mM KH_2_PO_4,_ 500 mM NaCl, 20% Glycerol, 0.1 mM PMSF, 20 mM Imidazole, 10 mM β-ME, pH: 7.5 (HCl). The sonicated mixture was centrifuged at 10,000 rpm for 1 h at 4 °C, and the subsequent supernatant was loaded onto a Ni–NTA column, which contains 1~2 mL Ni-agarose resin, and was rocked at 4 °C for 45~60 min. The His-tagged target protein was eluted using an imidazole concentration (200–500 mM). The eluted protein was analyzed by 10% SDS-PAGE. 

### 5.5. Enzyme Activity Measurement

#### 5.5.1. ZEA Degradation Optimization

For ZEA degradation, ZHDCP, ZHD101 and carboxypeptidase enzymes had chosen. The ZEA degradation test (in a total volume of 1 mL) was started by adding concentrated purified protein, where ZEA 5 ppm, 100-μg enzyme, 900-μL reaction buffer (150 mM NaCl, 25 mM Tris-HCl, pH: 9.5). The reaction was completed by heating the reaction mixture at 98 °C for 2 min. For identification of ZEA and its metabolite, the reaction mixture was filtered and analyzed by UV-HPLC (274 nm). The optimum pH was investigated at 30 °C in different buffers in the pH range 4-11. The optimum temperature was studied at pH 7.5 using temperatures ranging from 20 to 45 °C. The UV-HPLC running parameter deployed are as follows: Agilent Zorbax Extend-C18, 3 × 150 mm, 3.5 μm particle size); mobile phase: methanol/water/formic acid (10/90/0.1, *v*/*v*/*v*), flow rate: 0.2 mL/min; nitrogen drying gas: 10 L/min, nebulizer pressure: 25 psi, drying gas temperature: 350 °C, capillary voltage: 4 kV.

#### 5.5.2. OTA Degradation Optimization

In preliminary studies, fusion (ZHDCP) enzyme and carboxypeptidase enzyme were identified for their capacity to degrade OTA at different temperature ranges (20, 25, 30, 35, 40, 45 °C) and pH region 4-11. The reaction mixture was incubated at 31 °C with gentle shaking for 0, 1, 2, 4, 12 and 24 h to determine the optimum biodegradation time. In shortly, a 1 mL mixture containing 100 μg enzyme and toxin 5 ppm in a reaction buffer (150 mM NaCl, 25 mM Tris–HCl, pH 7.5) was incubated at 30 °C for 10 min. The reaction was terminated by heating reaction at 98 °C for 2 min. The experimental controls included the following: (1) OTA with acetonitrile solution (at a final concentration of 5 µg mL^−1^) and (2) Buffer with OTA. The experiments were performed in triplicate. The remaining OTA was then analyzed by UV-HPLC (214 nm) apparatus. OTA detection by using an isocratic mobile phase of methanol/water/acetic acid (60:50:2) at 30 °C and a flow rate of 1 mL/min. Enzyme activity was evaluated and measured by employing an HPLC calibration curve for OTA and ZEA concentration versus peak area.

#### 5.5.3. Fourier Transform Mass Spectrometry

The appropriate atomic mass quantification and enzyme-degraded product structure was identified and elucidated by a Thermo Triple Quadrupole Mass Spectrometry (TSQ) Vantage mass spectrometer (Massachusetts, USA). The data procurement was carried out with help of SP2(2.2) thermo foundation software. The instrument condition and method run both positive and negative ionization mode for scan and identified real degradation product structure and orientation. The running parameter was followed according to Bittner et al., 2015 with slight modification [41].

### 5.6. In Vitro Cell Cytotoxicity Assay

#### 5.6.1. Cell Seeding Environment and Incubation Conditions

The human Embryonic Kidney (HK293) and human liver (LX2) cells were obtained from the American Type Culture Collection, ATCC (Manassas, VA, USA). The cells were cultivated in Dulbecco’s Modified Eagles Medium, DMEM (Gibco™, High Glucose, Thermo Fisher Co. Ltd., Beijing, China) containing 10% fetal bovine serum and 1% penicillin/streptomycin growing under a humid atmosphere of 5% CO_2_—95% air at 37 °C. LX2 cell is cultured in RPMI-1640 medium from Gibco Co Ltd. (Beijing, China). When the cells were reached to ∼80–90% confluence, the media were changed after washing twice with phosphate buffered saline and applied with trypsin containing 0.25% EDTA for ∼1 min until most of them detached from individual cell. The collected cells after centrifugation for 5 min at 800 rpm were dissolved in DMEM and proliferated to exact cell amounts based on the kind of investigation.

#### 5.6.2. Measurement of Cell Viability by CCK-8 Test

Cell Counting Kit-8 (CCK-8) (Dojindo Laboratories, Tokyo, Japan) test were performed by plating 1.2 × 10^4^ HK293/LX2 cells per well in a 96 well plate (Thermo Fisher Scientific, Inc., Beijing, China). After growth of 48 h, the cells were propagated in a serum-free medium for 24 h in order to exclude any binding of the assayed compounds to serum proteins. Then solutions of OTA and ZEA were added to the cells in a concentration range from 1 µM to 100 μM and incubated for 24 h. Cells with same amount of solvent concentration were incubated as a control. Afterward, the viability of the cells was measured as the addition of CCK-8 reagent for 2 h, and the absorbance of each well was detected using a microplate reader (Thermo Fisher Scientific, Inc, Waltham, MA, USA) at a wavelength of 450 nm. This experimental setup was simultaneously done with ZHDCP, CP enzyme, and their ZEA/OTA degradation final products for comparative study [41]. 

#### 5.6.3. Total RNA Extraction and Determination of qRT-PCR

LX2 cells were plated into 6-cm sterile petri dishes at a concentration of 3.0 × 10^5^ cells per seed well and hatched for 24 h. Then the culture medium was changed with new medium containing 50-μM ZEA, 50-μM OTA, and their final products and buffer (final concentration 0.1%) as the negative control. After 24 h, the entire mRNA of cells mass was obtained using Trizol reagent (RNA direct Zol Zymo) according to the company’s instruction. The mRNA was converted to cDNA by PrimeScript synthesis kit (Takara, Japan) and was amplified by quantitative Real-time polymerase chain reaction (qRT-PCR) using a Power-Up SYBR Green Master Mix (ABI, Thermo Fisher Scientific, Waltham, MA, USA) was used to measure the mRNA expression of apoptosis-related genes (Caspase3, Sirt1, Bax, IL6, TNFα, Bcl2), and β-Actin mRNA level was used as the internal control gene for calculating the gene fold changes. The sequences of the specific primers used were as follows in Table A1 and were collected from preserved National Center for Biotechnology Information (NCBI) GenBank nucleotide sequences, and they were synthesized by Sangon Biotech (Shanghai, China). RT-PCR was performed by a Quant Studio Real-Time PCR Q7 thermal cycler (Applied Biosystems, Foster City, CA, USA). The PCR reaction mixture and cycle was performed according to ABI standard protocol for Q7 system. The relative mRNA expression of the apoptosis kinin hormone mRNA was measured by the 2^−∆∆Ct^ method [42].

#### 5.6.4. Apoptosis Assay

Determination of in vitro cell apoptosis was achieved by Annexin V-FITC and propidium iodide [39] staining apoptosis detection commercial kit (BD Pharmingen™ Co. Ltd., San Diego, CA, USA), according to the producer’s guidelines. The cells were cultured into a 6 well plate at a concentration of 3.0 × 10^5^ cells per well and incubated for 24 h. Following the incubation, the culture medium was replaced with new medium having 50-μM ZEA, 50-μM OTA, and ZEA/OTA final products for another 24 h, where buffer (final concentration 0.1%) as the negative control acted as the well media. After 24 h, the cells were washed twice in ice-cold Phosphate Buffered Saline and harvested, then suspended in Annexin V-FITC binding buffer, stained with fluorescent isothiocyanate (FITC)-labeled Annexin V and PI at 37 °C for 15 min in the absence of light, and then measured by flow cytometry (Guava, easy cyte 12HT, 96-well plate reader) through observing 20,000 cells for each experimental result identification.

### 5.7. Statistical Analysis

Data are shown as the averages ± standard deviation of three parallel experiments. Statistical calculation was performed by using two-tailed unpaired Student’s t-tests for comparison of differences between two groups. Data were analyzed and plotted using GraphPad Prism 7 (GraphPad software Inc, San Diego, CA, USA). Differences between treatments were considered to be significant for p-values less than 0.05.

## Figures and Tables

**Table 1:** Optimization of enzyme activity for ZEA and OTA degradation rate in terms of pH.

pH	ZEA			OTA	
	ZHDCP (%)	ZHD (%)	CP (%)	ZHD-CP (%)	CP (%)
**4**	10.2 ± 3.8	19.7 ± 1.9	13.9 ± 0.2	7.7 ± 0.7	0.3 ± 0.4
**5**	27.1 ± 2.0	27.1 ± 2.0	15.3 ± 1.5	13.6 ± 1.6	29.0 ± 0.7
**6**	54.7 ± 0.5	54.7 ± 0.5	21.1 ± 1.0	23.5 ± 0.5	30.4 ± 1.1
**7**	56.5 ± 2.0	57.7 ± 0.4	39.8 ± 5.5	31.9 ± 1.1	34.4 ± 0.3
**8**	56.4 ± 4.6	65.2 ± 7.9	13 ± 3.2	20.2 ± 0.8	23.8 ± 0.8
**9**	55.5 ± 0.5	55.5 ± 0.5	6.7 ± 0.1	17.0 ± 3.7	15.0 ± 2.2
**10**	48.6 ± 5.6	55.5 ± 0.5	4.2 ± 1.2	23.4 ± 2.1	9.3 ± 3.2
**11**	27.3 ± 1.7	10.2 ± 3.8	0.1 ± 0.1	26.3 ± 7.0	5.0 ± 0.6

Where, ZHD-CP fusion enzyme, ZHD: ZHD101 enzyme, CP: Carboxypeptidase enzyme, OTA: Ochratoxin A, ZEA: Zearalenone, ZEA 5 ppm, OTA 5 ppm, all enzymes are 30 µg/mL concentration. Incubation time 1 h for each treatment. All are three independent experiments and error bar represent mean ± SD.

**Table 2:** Optimization of enzyme activity for degradation of ZEA and OTA in term of temperature.

°C	ZEA			OTA	
	ZHDCP (%)	ZHD (%)	CP (%)	ZHD-CP (%)	CP (%)
**20**	47.8 ± 4.6	45.2 ± 4.0	32.0 ± 6.8	24.0 ± 5.2	9.6 ± 5.0
**25**	57.0 ± 6.3	48.3 ± 3.9	37.7 ± 7.8	37.9 ± 0.9	47.8 ± 7.5
**30**	65.4 ± 3.4	49.9 ± 0.4	53.5 ± 0.8	46.8 ± 0.1	51.7 ± 4.5
**35**	79.2 ± 0.4	54.6 ± 0.6	65.4 ± 5.4	45.6 ± 2.1	81.9 ± 13.2
**40**	66.8 ± 1.2	66.2 ± 1.4	57.6 ± 2.5	40.3 ± 5.4	63.7 ± 5.1
**45**	69.3 ± 2.4	58.4 ± 1.4	51.6 ± 1.0	38.4 ± 5.7	55.1 ± 7.1

Where, ZHDCP fusion enzyme, ZHD: ZHD101 enzyme, CP: Carboxypeptidase enzyme. OTA: Ochratoxin A, ZEA: Zearalenone. ZEA 2.5 ppm, OTA 2.5 ppm, all enzymes are 30 µg/mL concentration. Incubation time 1 h for each treatment. Data represent mean ± standard deviations of three independent experiments.

**Table 3:** ZEA and its derivate after degradation by fusion (ZHDCP) & Carboxypeptidase enzyme (LC/MS).

Final Product	HZEN 336.69	DZEN 292.38	α-Zearalenol 320.3802	β-Zearalenol 320.3802	α-Zearalanol 322.40	β-Zearalanol 322.40
ZHDCP ZEA Final products	Detect	Detect	N/D	N/D	N/D	N/D
Carboxypeptidase ZEA Final products	Detect	Detect	N/D	N/D	N/D	N/D

Where, N/D: non detected.

## Appendix A

**Table A1:** Primers and vector used in this study.

Gene	Accession No.	Primer	5′–3′ Sequence	Cloning Vector
Complementation primers				
ZHD101 Fusion	AB076037 (795)	Forward	GGAATTCATGCGCACCCGTAGTACCA (EcoRI)	pET28a
		Reverse	CAATCGGATCATTGCCGGCCAGGTGTTTCTGGGTGGTCTC	
Carboxy Fusion	KP161493 (1332)	Forward	GAGACCACCCAGAAACACCTGGCCGGCAATGATCCGATTG	pET28a
		Reverse	CCAAGCTTTTAGAACCAGCCGGTCACG (HindIII)	
ZHDCP sequence		Zhd101-seq	GGCATGCACTTTCCGTATGTG	
ZHD101	AB076037 (795)	Forward	GGAATTCATGCGCACCCGTAGTACCA (Ecor I)	pET28a
		Reverse	CCAAGCTTTTACAGGTGTTTCTGGGTGGTCTC (HindIII )	
Carboxy	KP161493 (1332)	Forward	GGAATTCATGGCCGGCAATGATCCGATTG (Ecor I)	pET28a
		Reverse	CCAAGCTTTTAGAACCAGCCGGTCACG (Hind III)	
Sequencing primer		M13-47	AGGGTTTTCCCAGTCACG	PMD18T
		M13-48	GAGCGGATAACAATTTCACAC	
Sequencing primer		T7-Pro	TAATACGACTCACTATAGG	pET28a
		T7-Ter	GCTAGTTATTGCTCAGCGG	
Real-time PCR primers				
Sirt1	NM_012238.4	Forward	CGGATTTGAAGAATGTTGGT	
		Reverse	ATCTGCTCCTTTGCCACTC	
Bax	NM_004324.3	Forward	CCGATTCATCTACCCTGCTG	
		Reverse	TGAGCAATTCCAGAGGCAGT	
β-Actin	NM_001101.3	Forward	CCTGGCACCCAGCACAAT	
		Reverse	GGGCCGGACTCGTCATAC	
Bcl-2	NM_000633.2	Forward	GAGGATTGTGGCCTTCTTTG	
		Reverse	GTGCCGGTTCAGGTACTCA	
TNF-α	NM_000594.3	Forward	CTTCTGCCTGCTG CACTTTGGA	
		Reverse	TCCCAAAGTAGACCTGCCCAGA	
IL-6	NM_000600	Forward	AGACAGCCACTCACCTCTTCAG	
		Reverse	TTCTGCCAGTGCCTCTTTGCTG	
Caspase-3	NM_214131.1	Forward	GACACTCGCTCAACTTCTTGG	
		Reverse	TTGGACTGTGGGATTGAGAC	
